# Integrated analysis of lncRNA and mRNA repertoires in Marek’s disease infected spleens identifies genes relevant to resistance

**DOI:** 10.1186/s12864-019-5625-1

**Published:** 2019-03-28

**Authors:** Zhen You, Qinghe Zhang, Changjun Liu, Jiuzhou Song, Ning Yang, Ling Lian

**Affiliations:** 1 Department of Animal Genetics and Breeding, College of Animal Science and Technology, China Agricultural University, Beijing, 100193 China; 2 Division of Avian Infectious Diseases, Harbin Veterinary Research Institute of Chinese Academy of Agricultural Sciences, Harbin, 150001 China; 3 Department of Animal & Avian Sciences, University of Maryland, College Park, MD 20742 USA

**Keywords:** Chicken, Long noncoding RNA, Marek’s disease, MD resistance

## Abstract

**Background:**

Marek’s disease virus (MDV) is an oncogenic herpesvirus that can cause T-cell lymphomas in chicken. Long noncoding RNA (lncRNA) is strongly associated with various cancers and many other diseases. In chickens, lncRNAs have not been comprehensively identified. Here, we profiled mRNA and lncRNA repertoires in three groups of spleens from MDV-infected and non-infected chickens, including seven tumorous spleens (TS) from MDV-infected chickens, five spleens from the survivors (SS) without lesions after MDV infection, and five spleens from noninfected chickens (NS), to explore the underlying mechanism of host resistance in Marek’s disease (MD).

**Results:**

By using a precise lncRNA identification pipeline, we identified 1315 putative lncRNAs and 1166 known lncRNAs in spleen tissue. Genomic features of putative lncRNAs were characterized. Differentially expressed (DE) mRNAs, putative lncRNAs, and known lncRNAs were profiled among three groups. We found that several specific intergroup differentially expressed genes were involved in important biological processes and pathways, including B cell activation and the Wnt signaling pathway; some of these genes were also found to be the hub genes in the co-expression network analyzed by WGCNA. Network analysis depicted both intergenic correlation and correlation between genes and MD traits. Five DE lncRNAs including MSTRG.360.1, MSTRG.6725.1, MSTRG.6754.1, MSTRG.15539.1, and MSTRG.7747.5 strongly correlated with MD-resistant candidate genes, such as IGF-I, CTLA4, HDAC9, SWAP70, CD72, JCHAIN, CXCL12, and CD8B, suggesting that lncRNAs may affect MD resistance and tumorigenesis in chicken spleens through their target genes.

**Conclusions:**

Our results provide both transcriptomic and epigenetic insights on MD resistance and its pathological mechanism. The comprehensive lncRNA and mRNA transcriptomes in MDV-infected chicken spleens were profiled. Co-expression analysis identified integrated lncRNA-mRNA and gene-gene interaction networks, implying that hub genes or lncRNAs exert critical influence on MD resistance and tumorigenesis.

**Electronic supplementary material:**

The online version of this article (10.1186/s12864-019-5625-1) contains supplementary material, which is available to authorized users.

## Background

Long noncoding RNA (lncRNA) is a class of noncoding RNAs with sequences longer than 200 nucleotides that are unable to translate into functional proteins. The nature of lncRNAs has been well characterized in mammals. LncRNAs are shorter in length, have fewer exons, and exhibit lower expression levels and less evolutionary conservation compared with protein coding genes [1–3]. Advances in sequencing technology afford extensive insight into genomic structure. Many lncRNAs have been discovered by RNA sequencing (RNA-Seq), which provides a robust technology for the comprehensive analysis of transcription at single-nucleotide resolution with superior depth [4–7]. It has been demonstrated that lncRNAs are relevant to biological processes and cellular development, especially in the disease state [8–12]. Mutations as well as the aberrant expression of functional lncRNAs may induce various diseases and biological disorders. For example, overexpression of lncRNA HOTAIR can result in breast cancer metastasis [13]. It has been reported that certain annotated lncRNAs have the ability to encode small peptides [14–16].

Marek’s disease (MD) is a complex immunosuppressive disease. It can cause paralysis, neuroinflammation, and chronic depletion, as well as lymphomas in chicken viscera and muscle tissue [17, 18]. MD is caused by Marek’s disease virus (MDV), a double-stranded DNA α-herpesvirus. The criteria for assessing the virulence of MDV have been established and the toxicity is ranked from mildly virulent (m), virulent (v), very virulent (vv) to very virulent plus (vv+) [19]. Incursion of the strains v and vv will cause transient paralysis in most breeds, whereas vv + strains will cause chicken brain lesions and eventually lead to death [20]. More seriously, as the virus evolves, its virulence is gradually strengthening [19]. The infectious life cycle of MDV in susceptible chicken lines can be divided into four stages: (1) establishment of primary infection; (2) semi-productive lytic viral replication in lymphocytes; (3) immune evasion and latency; and (4) tumor metastasis stage [21]. MDV has proven to be a valuable model virus for studying several human diseases caused by other herpesviruses; moreover, the MDV-chicken system also gives us a highly available and efficacious model to understand virus-induced lymphomagenesis. As tumor formation occurs only a few weeks after infection with different MDV strains in chicken lines, it is possible to easily perform herpesvirus-induced oncogenesis studies in chickens [22].

In recent years, more and more lncRNAs have been discovered and their functions revealed. However, research on lncRNAs in domestic animals is very limited. Of all noncoding transcripts in various animal species, the transcriptomes in domestic animals are inadequately characterized compared to human and model organisms [23]. Therefore, the discovery and functional annotation of lncRNAs in domestic animals is overdue. Several studies have revealed that lncRNAs play important roles in improved productivity in chickens [24–29]. Research on the roles that lncRNAs play in diseases resistance, particularly in MD, remain limited in chickens [30, 31].

In this study, the comprehensive transcriptomes of spleen tissues from 17 chickens with different MD resistance were analyzed. To identify candidate lncRNAs associated with MD resistance, integrated repertoires of lncRNAs and mRNAs of the spleen and their expression patterns were profiled. Differential expression and co-expression network analysis were conducted to identify interactions between mRNAs and lncRNAs with regard to their underlying roles in resisting tumorigenesis at the late neoplastic transformation stage of MDV infection.

## Results

### Identification of lncRNAs

**Fig. 1:**
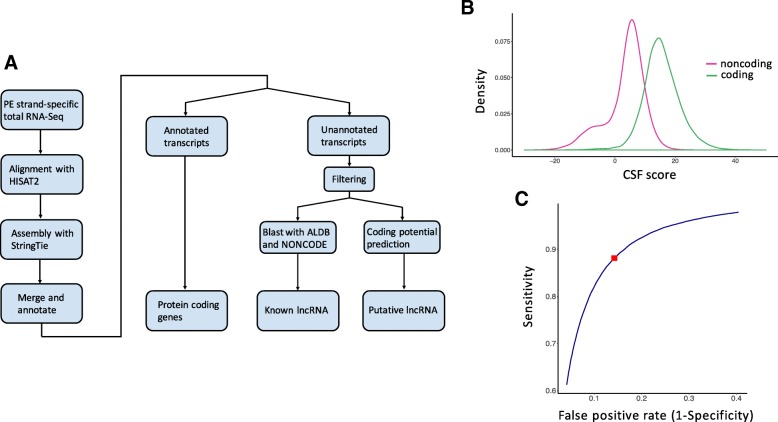
Overview of lncRNA identification in chicken spleen. **a** an overview of a comprehensive scheme for the identification of lncRNAs in chicken spleen. **b** the distributions of CSF scores of both coding and noncoding training data sets in CSF classifier. **c** receiver operating characteristic (ROC) analysis for training data sets to set a CSF score cut-off. The area under the curve (AUC) is 0.94 and the red square in line represents the optimal point with the best sensitivity and specificity

A total of 17 samples were used for this study, including seven tumorous spleens (TS) and five spleens of survivors (SS) from MDV-infected chickens, and five noninfected spleens (NS) from mock infected chickens. In all, 275.5 gigabytes (GB) of RNA-Seq data sets were analyzed and 273.477 GB remained after eliminating the low-quality reads. In order to explore putative lncRNAs from the chicken splenic transcriptome, a lncRNA identification pipeline was designed (Fig. 1a). Clean reads were aligned to the chicken reference genome (Gallus_gallus-5.0) and reads not properly mapped were discarded, resulting in overall mapping rates of 84.57 to 92.55% (Table 1). Finally, 16,682 unannotated transcripts (28.29% of the total transcripts from all samples) remained. Among unannotated transcripts, we identified 1166 known lncRNAs through aligning with the lncRNAs either in NONCODE v5.0, an integrated non-coding RNA database, or in ALDB, a domestic-animal long noncoding RNA database (Additional file 1).

**Table 1:** Information on the results of RNA-Seq data quality control and mapping

Sample ID	Raw reads	Clean reads	Read length	Mapped reads	Concordantly mapped pairs	Concordant pair align rate (%)	Overall alignment rate (%)	Depth of coverage	Coverage
s17–1	108,038,362	107,236,056	150	99,071,070	47,355,310	88.32	92.39	71.44	99 M
s17–2	104,641,540	104,150,474	150	95,831,146	45,554,825	87.48	92.01	69.72	96 M
s14–5	104,552,356	104,012,122	150	96,002,848	45,706,899	87.89	92.3	59.93	96 M
s21–5	115,513,772	114,724,610	150	105,871,206	50,359,014	87.79	92.28	71.65	106 M
s12–1	121,782,278	121,124,168	150	111,411,790	53,080,305	87.65	91.98	72.78	111 M
s10–4	95,689,198	94,726,246	150	84,233,771	39,193,992	82.75	88.92	51.00	84 M
s13–2	102,265,142	100,720,338	150	89,382,799	41,657,926	82.72	88.74	59.45	89 M
s14–8	92,679,650	91,878,888	150	81,576,308	38,013,902	82.75	88.79	43.88	81 M
s16–2	100,808,994	100,163,884	150	92,596,205	44,222,526	88.3	92.44	60.53	93 M
s6–3	106,793,466	105,995,894	150	94,836,775	44,425,877	83.83	89.47	71.91	95 M
s7–2	117,484,294	116,319,496	150	98,372,279	45,819,569	78.78	84.57	66.10	98 M
s9–2	101,533,692	100,601,162	150	87,682,429	40,550,892	80.62	87.16	51.14	88 M
s24–11	108,771,468	108,057,900	150	100,009,407	47,516,011	87.95	92.55	54.06	100 M
s24–6	117,780,440	117,111,392	150	107,874,786	51,288,711	87.59	92.11	54.88	108 M
s24–7	125,499,128	124,725,286	150	114,662,567	54,498,218	87.39	91.93	72.64	115 M
s24–8	112,133,382	111,530,648	150	103,004,010	49,103,238	88.05	92.35	70.26	103 M
s24–9	100,700,518	100,087,486	150	92,311,210	43,884,783	87.69	92.23	55.25	92 M

Besides known lncRNAs, 1653 transcripts were identified as putative lncRNAs by CPC, CNCI, and PLEK. Next, we applied the codon substitution frequency (CSF) algorithm using custom python scripts for the second round of novel lncRNA filtration. Standard CSF scores of both coding and non-coding sequences were obtained from training data sets (Fig. 1b; see Methods for CSF in detail). According to the receiver operating characteristic (ROC) curve, the area under the curve (AUC) is 0.94, indicating that the CSF score is a good classifier for estimating the coding potential of unannotated sequences. As the CSF score corresponding to the best classification point in ROC curve was 9.8, transcripts with CSF scores less than 9.8 were marked as lncRNAs (Fig. 1c). Robustness test for the CSF classifier was performed on randomly selected protein coding genes and lncRNAs in chicken. The results revealed that 724 genes out of 785 Ensembl chicken coding sequences (CDSs) were correctly marked as protein coding genes and 1893 lncRNAs out of 1942 NONCODE chicken lncRNAs were correctly marked as noncoding genes. The false discovery rate was 7.8% and the sensitivity was 97.5%. This confirms that the CSF score is a reliable classifier in distinguishing chicken lncRNAs from mRNAs. As a result, 1315 putative lncRNAs were ultimately obtained (Additional file 2).

### Genomic features of putative lncRNAs

**Fig. 2:**
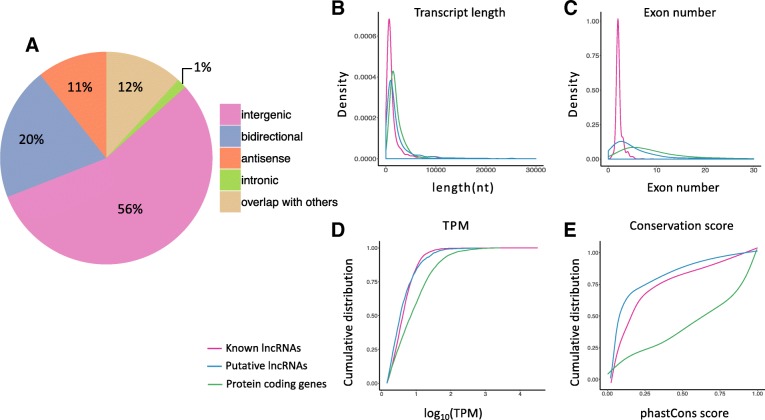
Genomic features and classification of putative lncRNAs. **a** classification of putative lncRNAs according to their genomic position in protein coding genes. **b** distribution of transcripts length. **c** distribution of exon number of lncRNAs and mRNAs. **d** cumulative distribution curve of gene expression levels (TPM). **e** cumulative distribution of conservation scores

We further analyzed the length, exon number, expression abundance, and evolutionary conservation of the putative lncRNAs by comparing them with protein coding genes and known lncRNAs. The majority of putative lncRNAs were intergenic (56%), 20% were bidirectional, 11% were antisense, and 1% were intron lncRNAs (Fig. 2a). The distributions of putative lncRNAs on the chromosomes varied. Over half of putative lncRNAs were located on the Z chromosome (~ 10%) and the incompletely assembled scaffolds (~ 48.7%) (Additional file 3: Figure S1). The lengths of the putative lncRNAs were shorter than those of the protein coding genes, but longer than the known lncRNAs (*P*-value < 1 × 10^− 6^, Welch’s t-test). The exon numbers of the putative lncRNAs were less than the protein coding genes while greater than the known lncRNAs (*P*-value < 1 × 10^− 6^, Welch’s t-test). Most of the chicken lncRNAs in the current ALDB and NONCODE databases are partially assembled. These results reflect that the putative lncRNA in our study have greater integrity than previously assembled lncRNAs, due to the better sequencing depth and coverage. While both novel lncRNAs and known lncRNAs exhibited lower expression abundance compared to the protein coding genes (*P*-value < 1 × 10^− 6^, Welch’s t-test), there was no significant expressional difference between putative and known lncRNAs (P-value = 0.19). In terms of evolutionary conservation, the putative lncRNAs were significantly less conserved than the protein coding genes (P-value < 1 × 10^− 6^, Welch’s t-test) and the known lncRNAs (P-value = 7 × 10^− 4^, Welch’s t-test) (Fig. 2b-e).

### Expression patterns of lncRNAs and mRNAs among three groups

**Fig. 3:**
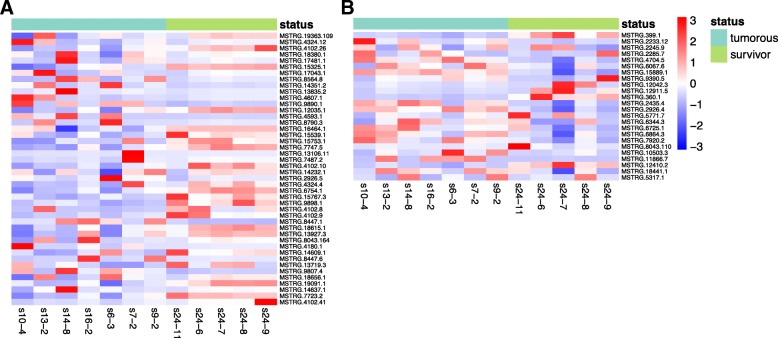
Heatmaps of differentially expressed lncRNAs between the tumorous group and the survivors. **a** the expression levels of DE putative lncRNAs in each individual from the tumorous spleens and the spleens of survivors. **b** DE known lncRNAs in each individual from the tumorous spleens and the spleens of survivors

R package DESeq2 provided us methods to test for differentially expressed (DE) protein coding genes and lncRNAs, including both known and putative lncRNAs. First, we focused on DE protein coding genes and lncRNAs in the TS versus SS contrast. Overall, 1604 DE genes were found, of which 564 were protein coding genes annotated by Ensembl. Among DE protein coding genes, 478 were upregulated in TS, while 86 were downregulated. Forty-four putative lncRNAs were found differentially expressed, of which 24 were upregulated and 20 were downregulated in TS (Fig. 3a). Regarding to known lncRNAs, 17 out of 24 were upregulated and 7 were downregulated in TS (Fig. 3b). The estimated power of the differential expression analysis in this contrast was 0.84. Second, we analyzed DE protein coding genes and lncRNAs in the TS vs. NS contrast, which revealed 2460 DE genes and 1180 of them were annotated as protein coding genes. Among the annotated genes, 1044 of them were upregulated and 136 were downregulated in TS. We found 56 DE putative lncRNAs, with 30 upregulated and 26 downregulated (Additional file 4: Figure S2A); 29 known lncRNAs were upregulated in TS, whereas 9 were downregulated (Additional file 4: Figure S2B). The estimated power of the differential expression analysis in this contrast was 0.89. Lastly, there were only five annotated protein coding genes, 22 putative lncRNAs, and 21 known lncRNAs that differentially expressed in the SS vs. NS contrast; most DE lncRNAs in this contrast overlapped with those in the other comparisons (Additional file 5). The estimated power of the differential expression analysis in this contrast was 0.85.

By comparing DE protein coding genes and lncRNAs in each contrast, we identified specific intergroup DE protein coding genes that may account for the different tumor incidence rates between TS and SS. There were 30 protein genes that were uniquely downregulated in TS vs. SS contrast, i.e., that were not identified as DE protein coding genes in the TS vs. NS contrast. Chickens in the TS and SS groups were all infected and SS chickens showed no clinical lesions 55 days postinfection (DPI), indicating the robust immune systems of the survivors. The interleukin receptor gene interleukin 1 receptor accessory protein like 1 (IL1RAPL1) and tumor-related gene tumor protein D52 like 1 (TPD52L1) were included in these 30 genes. B cell CLL/lymphoma 11A (BCL11A), being essential for lymphoid cell development, also was differentially expressed in the TS vs. SS contrast exclusively. In contrast, there were 110 protein genes that were upregulated merely in the TS vs. SS contrast, such as Wnt family member 10A (Wnt10A).

Furthermore, 83 protein coding genes were solely downregulated in the TS vs. NS contrast, including immune response related genes Wnt1 inducible signaling pathway protein 1 (WISP1), V-set pre-B cell surrogate light chain 3 (VPREB3), C-X-C motif chemokine ligand 12 (CXCL12), and some B cell activation genes CD79B molecule (CD79B), switching B cell complex subunit (SWAP70), and cholinergic receptor nicotinic alpha 7 subunit (CHRNA7), which implied host adaptive immunity deficiency in the tumorous individuals. Previously reported MD resistance-related genes, tetraspanin 8 (TSPAN8), toll like receptor 7 (TLR7), and histone deacetylase 9 (HDAC9), were also among 83 downregulated genes. Besides the specific downregulated genes, 676 protein coding genes were upregulated in the TS vs. NS contrast exclusively, including C-X-C motif chemokine ligand 14 (CXCL14), interferon gamma (IFNG), insulin like growth factor 2 mRNA binding protein 1 (IGF2PB1), hyaluronan synthase 3 (HAS3), and Wnt family member 7B, 8C, 8A, 11, and 11B (Wnt7B, Wnt8C, Wnt8A, Wnt11, and Wnt11B). Some interleukin precursor genes including interleukin 3, 9, and 13 (IL3, IL9, IL13) were also in this list.

### Co-expression analysis of lncRNAs and mRNAs

**Fig. 4:**
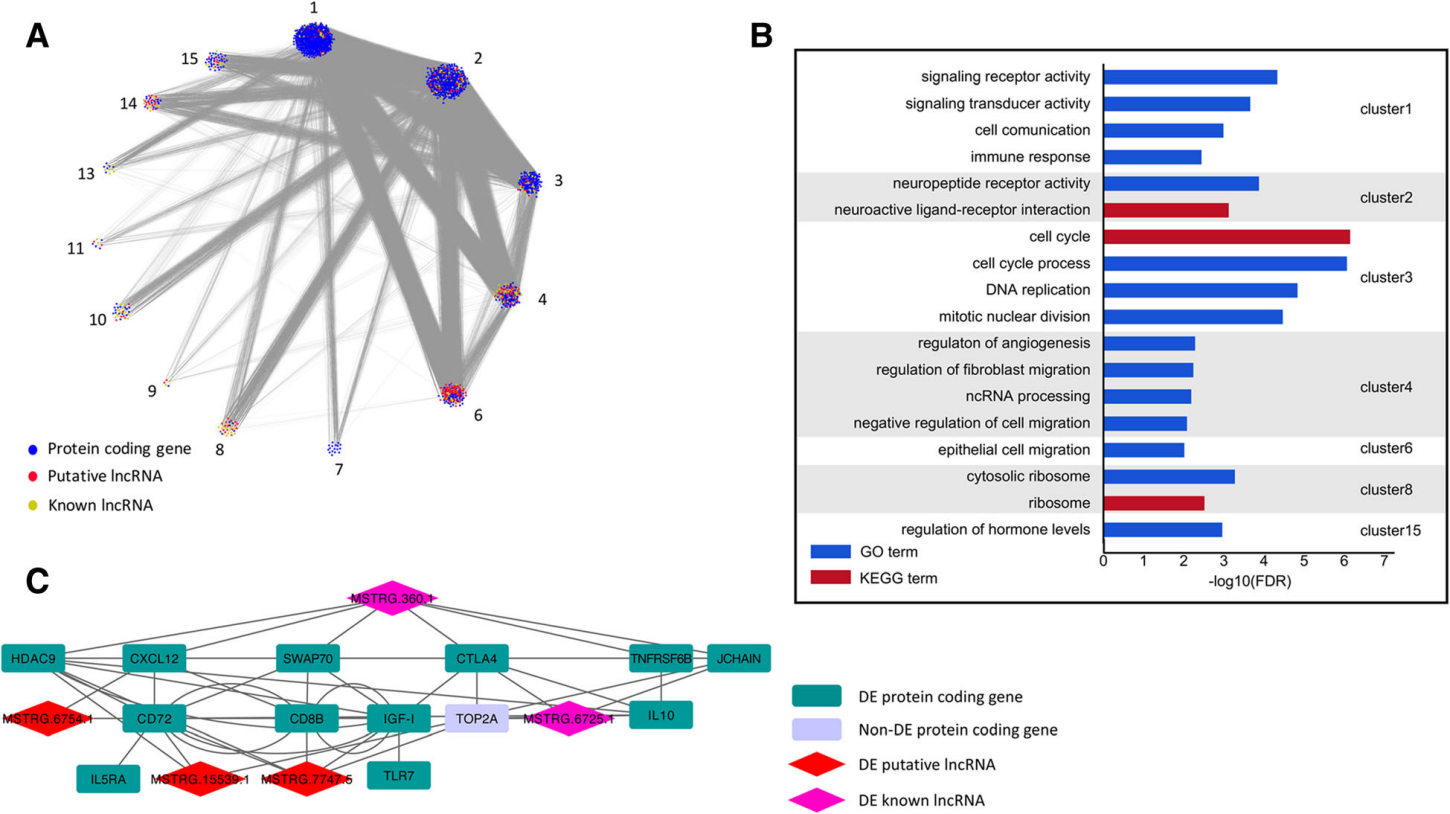
Co-expression network of DE protein coding genes and lncRNAs to predict the functional roles of candidate lncRNAs. **a** co-expression network visualization: nodes represent genes with the top 5% mutual correlation and each edge represents their close connection. **b** GO and KEGG pathway enrichment analysis for seven clusters with high phenotypic correlation. **c** sub-network of hub genes and candidate lncRNAs in cluster 1. This describes the relationship between candidate lncRNAs and hub protein coding genes

We constructed a co-expression network of lncRNAs and protein coding genes to infer the underlying regulatory function and potential target genes of DE lncRNAs (see Methods). Correlated genes and lncRNAs were grouped into 15 clusters by WGCNA and each cluster contained at least 50 genes. For all lncRNAs and protein coding genes in the co-expression network, the top 5% of highly correlated gene pairs according to the topological overlap matrix (TOM) were chosen to visualize the correlation of clusters (Fig. 4a). In this co-expression network, the highly correlated genes were grouped and the edges connecting two nodes indicated the functional regulatory relationship between the genes. Based on the correlation between the clusters and phenotypes, seven clusters including cluster 1, cluster 2, cluster 3, cluster 4, cluster 6, cluster 8, and cluster 15, were notably closely correlated with phenotype (Additional file 6).

Functional enrichment in each cluster suggested that lncRNAs and protein coding genes were functionally correlated with each other in specific biological processes. Annotated genes in cluster 1, where the largest proportion of DE lncRNAs located, were significantly enriched in signal transduction, intercellular communication, and immune response, suggesting that DE lncRNAs in cluster 1 appear to be essential for those biological processes, while protein coding genes in cluster 3 were significantly enriched in cell cycle and DNA replication. Protein coding genes in cluster 4 were significantly enriched in cell migration and non-coding RNA generation (FDR ≤ 0.01) (Fig. 4b).

Using WGCNA, we calculated the gene significance (GS) and module membership (MM) for every protein coding gene and lncRNA (Additional file 7). GS represented the correlation between genes and traits, and the MM represented the correlation between genes and each cluster. The protein coding genes or lncRNAs characterized by high GS and MM values (|GS| > 0.7, |MM| > 0.7) within clusters were regarded as hub genes, which reflected the main functions of the clusters and had strong correlation with phenotypes and other genes. Several DE genes in cluster 1 had high absolute values of GS and MM, including interleukin 10 (IL10), TNF receptor superfamily member 6b (TNFRSF6B), joining chain of multimeric IgA and IgM (JCHAIN), SWAP70, and CD72 molecule (CD72). In particular, specific intergroup DE genes such as HDAC9, cytotoxic T-lymphocyte associated protein 4 (CTLA4) and insulin like growth factor (IGF-I) had high GS and MM values as well. In cluster 3, in which annotated genes enriched in cell cycle regulation, Mov10 RISC complex RNA helicase (MOV10) was the hub gene in spite of no differential expression in each comparisons. These hub protein coding genes may play singularly functional roles in MD resistance and tumorigenesis.

Among 15 clusters, five DE lncRNAs with high GS and MM values were the most connected to many immune response-related and cell cycle-related DE genes. These five lncRNAs were all from cluster 1. Three of them were putative lncRNAs (lncRNA ID: MSTRG.6754.1, MSTRG.7747.5, and MSTRG.15539.1) and the others were known lncRNAs (MSTRG.6725.1 and MSTRG.360.1). The genomic locations of three putative lncRNAs are shown in Additional file 8: Figure S3. Of the five candidate DE lncRNAs, MSTRG.6754.1, MSTRG.7747.5, MSTRG.15539.1, and MSTRG.360.1 were downregulated in the TS group compared with the SS and NS groups, yet MSTRG.6725.1 was upregulated in the TS group.

A subnetwork of hub genes in cluster 1 was depicted in detail (Fig. 4c). Protein coding genes that were most connected to five candidate lncRNAs were likely the potential target genes of these lncRNAs. Known lncRNA MSTRG.360.1 (NONCODE ID: NONGGAT000276.2) was most correlated with TNFRSF6B, HDAC9, CTLA4, CXCL12, SWAP70, and JCHAIN; known lncRNA MSTRG.6725.1 (NOCODE ID: NONGGAT004747.2) was most correlated with CTLA4 and JCHAIN. Putative lncRNA MSTRG.6754.1 was most correlated with IGF-I, CD72, and CXCL12; putative lncRNA MSTRG.15539.1 was most strongly correlated with HDAC9, SWAP70, JCHAIN, and CD72; putative lncRNA MSTRG.7747.5 was most strongly correlated with HDAC9, CD8B molecule (CD8B), CD72, and IGF-I. In addition, known lncRNA MSTRG.360.1 was also highly correlated with MOV10 (correlation = − 0.92, *P*-value < 10^− 6^), suggesting that MSTRG.360.1 may have effect on mRNA cleavage and miRNA silencing. Notably, the DE protein coding genes CD72, CTLA4, HDAC9, JCHAIN, and SWAP70 in cluster 1 were also strongly correlated with each other, providing us with an integrated lncRNA-mRNA co-expression network.

Protein coding genes both as hub genes and as specific intergroup DE genes, e.g., CTLA4, HDAC9, CXCL12, JCHAIN, CD72 and SWAP70, must contribute tremendously to the modulation of host immunity during MD pathogenesis. The hub genes SWAP70 and CD72 that shared high correlation with the five candidate lncRNAs, along with specific intergroup DE genes BCL11A, VPREB3, CD79B, and CHRNA7 were all engaged in B cell proliferation and activation, highlighting that these DE genes acted as necessary regulatory factors for B cell functions in the late-stage humoral immunity of MDV infection and the five candidate lncRNAs likely involved in these functions by regulating SWAP70 and CD72. In addition, several specific intergroup DE genes were found in the Wnt gene family. WISP1 was the hub gene in cluster 4; it was most connected to candidate lncRNAs MSTRG.6754.1 (correlation = 0.81, *P*-value = 7.6 × 10^− 5^) and MSTRG.360.1 (correlation = 0.83, *P*-value = 3.7 × 10^− 5^). Inverse correlation between WISP1 and other members of the Wnt gene family [WIF1 and WISP1 pair (correlation = − 0.70, P-value = 1.8 × 10^− 3^), WISP1 and Wnt11 pair (correlation = − 0.75, P-value = 5.0 × 10^− 4^), and WISP1 and Wnt10A pair (correlation = − 0.80, P-value = 1.1 × 10^− 4^)] suggested that WISP1 negatively controlled the expression of these genes and candidate lncRNAs MSTRG.6754.1 and MSTRG.360.1 regulated the Wnt signaling pathway by controlling WISP1. DNA topoisomerase II alpha (TOP2A), a non-differentially expressed gene with high GS (0.87) and MM (− 0.97) values, was strongly correlated with CTLA4 and IL10 as well as two candidate lncRNAs MSTRG.7747.5 and MSTRG.6725.1. Though the expression level of TOP2A was not significantly different among groups based on DESeq2 method, it still exhibited substantial variance as calculated by MAD (Median Absolute Deviation). Furthermore, MSTRG.7747.5, which strongly correlated with both IGF-I (correlation = 0.94, P-value < 10^− 6^) and TOP2A (correlation = − 0.87, P-value < 10^− 6^), likely participated in the regulation of cell cycle.

### qPCR validation

**Fig. 5:**
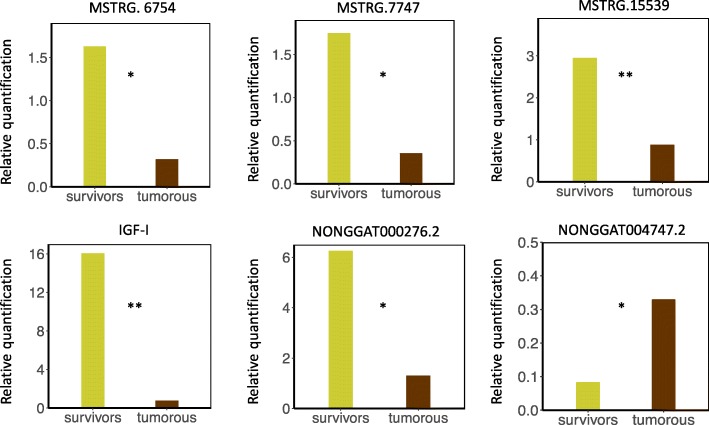
Quantitative PCR analysis of five candidate lncRNAs and IGF-I. * and ** respectively represent the significant difference in gene expression between two groups (* for *P*-value < 0.05 and ** for *P*-value < 0.01)

The expression levels of five candidate lncRNAs and DE gene IGF-I were confirmed by quantitative reverse transcription PCR (RT-qPCR) (Fig. 5). β-actin was used as the endogenous control. Total cDNA of 12 individuals in the TS and SS groups was used for quantitative analysis. The results of RT-qPCR were consistent with the results of RNA-Seq. Primer sequences and agarose gel electrophoresis pictures of five candidate lncRNAs are listed in Additional file 9.

## Discussion

In this study, we performed rRNA-free strand-specific transcriptome sequencing on all 17 samples and developed a precise pipeline to identify known and putative lncRNAs in chicken spleens. The use of paired-end, high-throughput sequencing ensured the integrity of the transcriptomes, making it possible to construct a more complete landscape of both known and novel lncRNAs. The lncRNAs obtained previously were poorly assembled. We hereby used an alignment method and set strict filtration parameters to identify known lncRNAs and re-assembled them comprehensively. A combination of two well-tested algorithms greatly reduced false positive rates in discriminating putative lncRNAs from unannotated transcripts. We characterized the genomic features of these lncRNAs and found that the results were in good agreement with previous studies, demonstrating the reliability of the putative lncRNAs we identified [26, 28, 31]. To better understand the underlying functional relationship between lncRNAs and mRNAs in tumorigenesis and MD resistance, we profiled comprehensive gene expression patterns in chicken spleen and constructed a co-expression network of lncRNAs and mRNAs.

### Specific intergroup DE genes in MD resistance and tumorigenesis

Many studies have analyzed changes in gene expression levels during different stages of MDV pathology in different chicken viscera. Most of them were interested in the cytolysis or latency stages. Given that MD is a complex disease—the pathogenicity of which cannot be explained by only a few genes—numerous genes were found bearing close relationships with MD resistance. By comparing previous studies, we found some DE protein coding genes overlapped with former results (Table 2). The expression levels of several MD resistant genes such as granzyme A (GZMA), IFNG, IL10, TPD52L1, IGF-I, and CXCL12 were significantly different between the tumorous spleens and the spleens of survivors, which was in line with our former microarray data described by Lian et al. [32]. More extensively, we found that some of them were specific intergroup differentially expressed. According to the co-expression network and considering its critical effects on the development of lymphocytes and monocytes, CXCL12, a hub gene in cluster 1—simultaneously strongly related to many other MD resistant protein coding genes, e.g., CD72 and CD8B, as well as candidate lncRNAs MSTRG.360.1 and MSTRG.6754.1—may play an important role in immune response and tumor growth and metastasis induced by MDV infection [33]. Yu et al. (2011) reported that CTLA4, HAS3, WISP1, and TSPAN8 was differentially expressed between MD-resistant and -susceptible chickens at 10 DPI [34]. These genes exclusively differentially expressed in TS vs. NS contrast at late neoplastic transformation phase, which indicated that these genes were transcriptionally varied between the TS and SS group at late neoplastic stage. There were very few DE annotated genes in the SS vs. NS contrast and we did not find DE lncRNAs in this contrast with high GS and MM values or strong correlation with MD resistance-related genes.

**Table 2:** Fold change and adjusted P-value of partial specific intergroup DE genes and MD resistanc-associated genes reported previously

Ensembl ID	Gene Name	TS vs. NS		TS vs. SS	
		Fold change	Padj	Fold change	Padj
ENSGALG00000000892	IL10	6.70	7.77 × 10^−18^	3.30	1.80 × 10^−3^
ENSGALG00000008267	IL5RA	−3.03	1.90 × 10^−5^	–	–
ENSGALG00000016288	IL1RAPL1	–	–	−3.44	1.29 × 10^−6^
ENSGALG00000021113	IL3	3.43	1.87 × 10^−4^	–	–
ENSGALG00000006329	IL9	4.62	2.17 × 10^−2^	–	–
ENSGALG00000006801	IL13	5.63	1.34 × 10^−3^	–	–
ENSGALG00000009903	IFNG	2.32	3.43 × 10^−4^	–	–
ENSGALG00000008666	CTLA4	3.04	3.15 × 10^−11^	–	–
ENSGALG00000014843	TPD52L1	–	–	−3.70	3.69 × 10^− 2^
ENSGALG00000030115	WISP1	−2.35	6.24 × 10^−4^	–	–
ENSGALG00000010152	TSPAN8	−2.68	4.69 × 10^−4^	–	–
ENSGALG00000016590	TLR7	−3.15	4.58 × 10^−7^	–	–
ENSGALG00000004096	CHRNA7	−2.42	9.81 × 10^−6^	–	–
ENSGALG00000005750	SWAP70	−2.02	7.08 × 10^−9^	–	–
ENSGALG00000015902	CD8B	−2.90	8.98 × 10^−6^	−3.37	6.70 × 10^−5^
ENSGALG00000005194	CD72	−3.32	4.91 × 10^−7^	−3.27	2.19 × 10^−4^
ENSGALG00000000251	CD79B	−2.69	4.97 × 10^−6^	–	–
ENSGALG00000010854	HDAC9	−2.26	9.44 × 10^−5^	–	–
ENSGALG00000000630	HAS3	5.24	3.11 × 10^−3^	–	–
ENSGALG00000006106	TNFRSF6B	10.88	1.11 × 10^−15^	2.78	2.22 × 10^−2^
ENSGALG00000006346	CXCL14	6.08	2.34 × 10^−3^	–	–
ENSGALG00000041346	CXCL12	−2.14	7.89 × 10^−5^	–	–
ENSGALG00000012755	IGF-I	−4.60	2.35 × 10^−7^	−4.15	6.40 × 10^−5^
ENSGALG00000041204	IGF2PB1	3.42	2.65 × 10^−4^	–	–
ENSGALG00000036519	CDC6	2.23	1.25 × 10^−2^	–	–
ENSGALG00000034048	BCL11A	–	–	−2.46	1.0 × 10^−2^
ENSGALG00000021136	VPREB3	−3.84	4.05 × 10^−5^	–	–
ENSGALG00000011355	Wnt10A	–	–	5.48	2.37 × 10^−2^
ENSGALG00000036255	Wnt7B	2.90	2.82 × 10^−2^	–	–
ENSGALG00000037494	Wnt8C	4.77	3.26 × 10^−2^	–	–
ENSGALG00000000839	Wnt11	4.48	1.72 × 10^−3^	–	–
ENSGALG00000004401	Wnt11B	3.77	8.98 × 10^−4^	–	–
ENSGALG00000039209	WIF1			3.78	6 × 10^−3^

Abbreviation: *TS* the tumorous spleens, *NS* noninfected spleens, *SS* the spleens of survivors. Fold change greater than zero represents that genes expression level increase in the tumorous spleens. Fold change smaller than zero represents that genes expression level decrease in the tumorous spleens. “-” means no difference in expression level between compared groups

We subsequently screened all specific intergroup DE genes that have not been reported previously. In these specific intergroup DE genes, BCL11A, VPREB3, CD79B, CHRNA7, and SWAP70 that all involved in B cell proliferation and activation, would likely impact B cell function in MDV pathogenesis and malignant tumor formation. Additionally, SWAP70 and CD72 were the hub genes in cluster 1. Some members of the Wnt gene family were specific intergroup DE genes, e.g., Wnt7B, Wnt8C, Wnt10A, Wnt11, and Wnt11B, as well as WIF1 (Wnt inhibitory factor 1) and cluster 4 hub gene WISP1. It has been reported that WISP1 is downstream of the Wnt signaling pathway and its overexpression will inhibit apoptosis and promote cell proliferation and migration in glioblastoma cells, as verified by a knockdown experiment in vivo [35]. However, other studies have revealed that WISP1 may be conducive as a proliferative agent [36, 37]. In our research, the reduction in expression levels of WISP1 was observed in the TS group compared with the NS group, which suggested that WISP1 may act as a tumor suppressor gene in MD-induced tumorigenesis, unlike its oncogenic role in many kinds of human cancers and cell lines.

### Interaction between candidate lncRNAs and hub genes

DE protein coding genes and lncRNAs in the TS group exhibited higher variability in expression levels compared with the SS and NS groups and no significant sex or time effects were observed in DE genes (Additional file 10: Figure S4). We conducted co-expression network analysis to find the relatedness between lncRNAs and mRNAs. Five candidate lncRNAs strongly correlated with the greatest number of immune-related and cell cycle-related genes and possessed high values of GS and MM. Most of their potential target genes were reported differentially expressed between MD-resistant and -susceptible chicken lines, e.g., HDAC9 and IGF-I. Some candidate lncRNAs shared the same potential target genes in functional regulation, such as CD72, SWAP70, CTLA4, and CXCL12, indicating the functional intersection of these lncRNAs and the importance of their common potential target genes. The expression variation of CD72, SWAP70 and other specific intergroup DE genes that affect B cell proliferation and activation in the host adaptive immunity system appeared to be the cause of B-cell function abnormality in the TS group. The strong correlation between five candidate lncRNAs and hub gene CD72 and SWAP70 highlighted the essential roles of lncRNAs in regulating B-cell function. Candidate lncRNAs MSTRG.6754.1 and MSTRG.360.1, strongly correlated with hub gene WISP1—the expression levels of which were inversely correlated with other members of the Wnt gene family, may inhibit malignant lymphoma formation through regulating Wnt signaling pathway. In addition, lncRNA MSTRG.7747.5 was highly correlated with cell cycle-related genes TOP2A and IGF-I. Our results showed that candidate lncRNAs were positively or inversely correlated to potential target genes, indicating that lncRNAs can either inhibit or facilitate gene expression. For instance, candidate lncRNA MSTRG.6725.1, upregulated in the TS group, was positively correlated with hub gene CTLA4 (correlation = 0.91, *P*-value < 10^− 6^), while inversely correlated with hub gene JCHAIN (correlation = − 0.89, P-value < 10^− 6^). Meanwhile, known lncRNA NONGGAT000276 produced two alternative splicing isoforms in our study, MSTRG.360.1 and MSTRG.360.2, and only MSTRG.360.1 differentially expressed between the TS vs. SS contrast and strongly correlated with MD resistance.

Given that researches investigating the function of long noncoding RNAs involved in the pathogenic mechanisms of MD in chicken are very limited, only two lncRNAs reported in previous MD studies compared with our data. The first was linc-Satb1 which were potentially expressed in bursa of an infected MD-resistant chicken line at 10 DPI; it strongly correlated with nearby protein coding gene SATB1 that was involved in T cell development and activation [31]. The second lncRNA was linc-Galmd3 reported by Han et al. (2017); they found that linc-Galmd3 was highly expressed in MDV-infected chicken CD4+ T cells in peripheral blood. A knockdown experiment revealed that loss of function of Galmd3 suppressed MD viral replication [30]. We aligned the sequence of these two lncRNAs with our transcripts and found they were 100% concordant in two known lncRNAs, MSTRG.5613 and MSTRG.9931, respectively, and the lengths of the two lncRNAs were longer than those of linc-Satb1 and linc-Galmd3, suggesting that MSTRG.5613 and MSTRG.9931 were likely the transcript isoforms of linc-Satb1 and linc-Galmd3 in chicken spleen. This may result from the difference between the two studies, including reference genome versions, sequencing depth, and the assembly algorithm. These two lncRNAs were not differentially expressed between comparisons. The likely reason may be the differences in tissue-types and MD pathological stages that we considered. The previous two studies focused on latency or early stages of MD pathogenesis while we were more interested in the late neoplastic stage; also, the viral strain we used was less virulent so that we could be certain the chicken would remain viable till the late neoplastic transformation.

## Conclusion

In this study, we presented splenic mRNA and lncRNA repertoires in chicken and found certain oncogenesis and MD resistance-related genes differentially expressed between groups. Through co-expression network analysis, we identified several hub genes that may play pivotal roles in MD resistance and tumorigenesis and also found five DE lncRNAs that were strongly related to these hub protein coding genes. Furthermore, one of the five lncRNAs plays a role in cell cycle regulation based on its close relationship with IGF-I and TOP2A. Several specific intergroup DE genes, as well as network hub genes, participated in B lymphocyte activation and the Wnt signaling pathway, e.g., BCL11A, SWAP70, WISP1, and Wnt11. The five candidate lncRNAs closely correlated with these protein coding genes, exerting significant effects on MD-induced tumorigenesis through regulating their target genes. We hope that the DE mRNAs and lncRNAs identified in this study provide valuable transcriptomic and epigenetic insights into MD resistance and its pathological mechanism.

## Methods

### Biological samples

Information on experimental samples was provided in our previous study [32]. Briefly, 150 one-day-old specific-pathogen-free (SPF) White Leghorn (BWEL) chicks were separated into two groups. One hundred of them were infected intraperitoneally with 2000 plaque-forming units (PFU) of the MDV-GA strain and the remaining birds were injected with the same volume of diluent (0.2 mL) as the noninfected group on the first day after hatching. The two groups were housed independently and they were observed 2–3 times daily. Birds were euthanized by T-61 intravenously (0.4 ml/kg) and tumorous spleens were collected. Animal experiments were approved by the Animal Care and Use Committee of China Agricultural University (Approval ID: XXCB-20090209) and the experiment was performed according to regulations and guidelines established by this committee. In our study, five noninfected spleens (NS), five normal spleens of the survivors (SS), and seven MDV-infected tumorous spleens (TS) were sampled, for which RNA-Seq was conducted. The collection time points and the gender of samples are shown in Table 3. Ribosomal RNA was removed by Epicentre Ribo-zero™ rRNA Removal Kit (Epicentre/Illumina, San Diego, CA, USA).

**Table 3:** Detailed information on 17 total samples

Group	Subgroup	Sample ID	Sex	Collection time point (dpi)
noninfected	–	s17–1	Female	43
noninfected	–	s17–2	Female	43
noninfected	–	s14–5	Male	40
noninfected	–	s21–5	Male	49
noninfected	–	s12–1	Female	37
infected	tumorous	s10–4	Female	35
infected	tumorous	s13–2	Female	38
infected	tumorous	s14–8	Female	40
infected	tumorous	s16–2	Female	42
infected	tumorous	s6–3	Female	31
infected	tumorous	s7–2	Male	32
infected	tumorous	s9–2	Female	34
infected	survivor	s24–11	Female	55
infected	survivor	s24–6	Female	55
infected	survivor	s24–7	Female	55
infected	survivor	s24–8	Female	55
infected	survivor	s24–9	Male	55

### RNA-Seq and transcriptome assembly

RNA-Seq were performed in all 17 samples using the Illumina Hiseq 4000 platform and 150 bp paired-end reads were generated. Ribosomal RNA was removed from total RNA prior to sequencing. RNA-Seq reads were qualified by NGS QC Toolkit [38]. Clean reads were aligned to the chicken reference genome using HISAT2 (version 2.1.0) and parameters were set as default [39]. Chicken reference genome and its annotation were downloaded from Ensembl (version Gallus_gallus-5.0; GCA_000002315.3). We used RSeQC to examine the rRNA residues [40]. We re-assembled the chicken transcriptome using StringTie (version 1.3.3b) [41]. The mapping and re-assembly were performed as previously described [42].

### Identification of lncRNAs

We used several strict filters to winnow potential lncRNAs from all transcripts. First, transcripts shorter than 200 nt and without strand information were removed; second, transcripts with class code “=”, “e”, “p,” and “c” were discarded; and third, transcripts with low expression levels (FPKM < 1) were filtered out. Subsequently, we downloaded the sequence of known lncRNAs from two multispecies lncRNA databases, ALDB (Domestic-Animal LncRNA Database) [43] and NONCODE [44], which contain 8923 and 12,850 chicken lncRNAs, respectively. Then we used BLAST (version 2.2.26) [45] to align the unannotated transcripts to lncRNAs from two databases with stringent parameters (e value ≤1 × 10^− 6^, perc_identity ≥90%, and alignment length must be longer than 90% of the length of lncRNAs from two databases). The transcripts perfectly aligned with sequences in either ALDB or NONCODE were regarded as known lncRNAs.

### Coding potential analysis

We calculated the coding potential of each transcript using three tools: CPC [46], CNCI [47], and PLEK [48]. CPC is considered a classic software for coding potential prediction. CNCI is less dependent on genomic information and is still robust in dealing with lncRNAs with much longer length. PLEK can handle long read-length sequences and is compatible with SNPs and short InDels. These three lncRNA predictor all based on the same classification model—support vector machine (SVM) and have proven to be highly effective in discriminating lncRNAs [49]. The intersection of the results from these three tools was used in the downstream analyses. We then calculated the CSF index of each unannotated transcript [50]. CSF is based on the frequency changes in the pairs of codons which are substituted inconsistently between coding and noncoding sequences in informants and target species. We chose human, mouse, rat, opossum, and zebra finch as informant species [51]. Multiple alignment was implemented using threaded-blockset aligner (TBA) [52]. When the pairs of codons met following conditions: (1) no gap in alignment; (2) no stop codons; (3) the aligned codons were not the same, then a CSF score would be assigned to the pair of codons. The score is a log likelihood ratio indicating how much more frequently substitution occurs in coding regions than in noncoding regions. This ratio is derived from the coding substitution matrix (CSM):

$$ {CSM}_{a,b}=P\left( informant\ codon\ b\ \right|\  target\ codon\ a,a\ne b\Big) $$

Given that the CSM of two training data sets were calculated, the standard coding sequences (CSM^C^) and the noncoding sequences (CSM^N^), the CSF score will be assigned to a codon substitution (a, b) by:

$$ \mathrm{CSF}=\mathit{\log}\frac{CSM_{a,b}^c}{CSM_{a,b}^n} $$

For coding sequences, we downloaded the CDSs of each annotated gene of the informant species and target species from Ensembl and then we randomly extracted 10,000 CDSs from them as the coding sequences training data set. For noncoding sequences, we randomly extracted 10,000 sequences from intergenic regions that were larger than 2 kB and contained no repetitive elements. For each codon substitution, the CSF score is calculated between the target species and each informant. CSF assigns a score to the alignment of target and each informant species by evaluating the score in every 90 bp sliding window, overlapping by 1 bp. The highest score out of these windows was the final score of the alignment. The median of CSF scores of each pair of codon from all informants was calculated to obtain a composite score. The CSF algorithm was implemented using custom python scripts. To determine the threshold of CSF scores from the training data set and to test whether the CSF score has a good classification efficiency, we performed ROC curve analysis on the CSF scores of both coding and non-coding sequences [53] and to set the CSF score threshold to classify coding and noncoding sequences. ROC curve analysis is an effective method to judge the quality of a classifier. According to the AUC calculated by the ROC curve, the larger the AUC, the better the classifier. The points with the best sensitivity and specificity on the ROC curve were selected and set as the best classification point. To further verify the robustness of CSF scores in our research, the CSF algorithm was conducted in 2727 sequences that were randomly selected, including 785 protein coding gene sequences from Ensembl and 1942 randomly selected chicken lncRNAs from NONCODE.

### Conservation analysis and differential expression analysis

Putative lncRNAs were categorized according to their genomic location as previously described [54]. We used phast software to compute the conservation score of protein coding genes, known lncRNAs, and putative lncRNAs [55]. Human, rat, mouse, opossum, and zebra finch were used as the queries. Fourfold degenerate sites were used to estimate the non-conserved model. The first codon positions were used to estimate the conserved model. After that, HMM transition parameters were tuned for phastCons to calculate the conservation score.

From all of the putative lncRNAs, known lncRNAs, and annotated protein coding genes, we used DESeq2 (version 1.16.1) to identify differentially expressed genes in different comparisons [56]. Read counts were fitted by generalized linear model and variables of sex and DPI were added in the design formula so that the variances caused by them would be corrected in differential expression analysis (design = ~ sex + dpi + status; where status represented tumorous, survivors, and noninfected). Protein coding genes and lncRNAs with differential expression levels must meet two criteria: adjusted *P* value < 0.05 (Benjamini-Hochberg adjustment) and |log2FoldChange| ≥ 2. Heatmap was drawn by R package pheatmap [57]. The power analysis was conducted using R package: RnaSeqSampleSize [58]. Powers were estimated in the TS vs. SS contrast, the TS vs. NS contrast, and the SS vs. NS contrast. Average reads count, ratio of geometric means of normalization factor, and median dispersion of DE genes were calculated by DESeq2 (estimateSizeFactors and estimateDispersions functions): 589, 1.1, 0.161 in the TS vs. SS contrast, 558, 1.08, 0.169 in the TS vs. NS contrast, and 673, 1.01, 0.116 in the SS vs. NS contrast, respectively.

### Co-expression network construction and functional enrichment analysis

An expression level matrix of all putative lncRNAs and known lncRNAs was constructed. In addition, given that genes without notable expression variation among samples would be highly correlated and influence the accuracy of the correlation network, the top 5152 most variant protein coding genes among the 17 samples were included and the median absolute deviation (MAD) was used as a variability measurement. We constructed a weighted co-expression network and calculated the Pearson correlation of each gene pair using R package WGCNA [59]. Hub genes were those that possessed high absolute values of GS and MM (both greater than 0.7) in their own clusters. The co-expression network was visualized using Cytoscape [60]. Highly correlated gene pairs (top 5% TOM) were listed as the input file for Cytoscape then the output network format was customized. Ensembl gene IDs were submitted to DAVID, a web-based functional enrichment analysis tool, to estimate enrichment in gene ontology (GO) terms and Kyoto Encyclopedia of Genes and Genomes (KEGG) terms [61]. The *P*-value of protein coding genes enrichment was adjusted by Benjamini-Hochberg FDR (false discovery rate) and its threshold was set as 0.05.

### Quantitative PCR

Total cDNA was synthesized and gDNA was removed using EasyScript One-Step gDNA Removal and cDNA Synthesis SuperMix (TransGen Biotech, Beijing, China). Power SYBR Green PCR Super Mix (Applied Biosystems, Foster City, CA, USA) was used as a nucleic acid stain and qPCR was performed on an ABI 7500 Real-Time PCR system. Chicken β-Actin was used as an endogenous control. Relative quantifications of genes were calculated by − 2^-ΔΔCT^ method. The primers were designed using NCBI Primer-Blast [62].

## Additional files

Additional file 1: Genomic location of 1166 newly re-assembled known lncRNAs in ALDB and NONCODE. (XLSX 196 kb)

Additional file 2: Genomic location and classification of 1315 putative lncRNAs. (XLSX 124 kb)

Additional file 3: **Figure S1.** Chromosomal distribution of 1315 putative lncRNAs. 641 putative lncRNAs (48.7%) located in small scaffolds which are not shown in this figure. Chromosome Z is the second-most putative lncRNAs which has 133 putative lncRNAs (10.1%). (PDF 5 kb)

Additional file 4: **Figure S2.** Heatmaps of DE lncRNAs between the tumorous spleens and noninfected spleens. **a** the expression level of DE putative lncRNAs in each individual from the tumorous spleens and noninfected spleens. **b** DE known lncRNAs in each individual from the tumorous spleens and noninfected spleens. (PDF 57 kb)

Additional file 5: DE protein coding genes, known lncRNAs and putative lncRNAs in 17 samples. (XLSX 237 kb)

Additional file 6: The number of protein coding genes and lncRNAs included in each clusters and the cluster-trait relationship. (XLSX 10 kb)

Additional file 7: Gene Significance, Module Membership, and *P*-value of protein coding genes and lncRNAs in WGCNA co-expression network. MM1 (Module Membership between gene and cluster 1); P_MM1 (P-value of each MM1); GS (Gene Significance); PG (P-value of each Gene Significance). (XLSX 3957 kb)

Additional file 8: **Figure S3.** Genomic location of three putative candidate lncRNAs and their overlapping elements. (PDF 26 kb)

Additional file 9: **Table S1.** Primer sets of five candidate lncRNAs and IGF-I used in quantitative PCR analysis; QPCR gel picture of five candidate lncRNAs; (DOCX 729 kb)

Additional file 10: **Figure S4.** Principal components analysis (PCA) of DE protein coding genes and lncRNAs in three comparisons, including DE putative lncRNAs, DE known lncRNAs, and DE protein coding genes. (PDF 64 kb)

## Abbreviations

AUC: area under the curve
CSF: codon substitution frequency
CSM: coding substitution matrix
dpi: day post-infection
GO: gene ontology
KEGG: Kyoto Encyclopedia of Genes and Genomes
MAD: median absolute deviation
MD: Marek’s disease
MDV: Marek’s disease virus
MHC: major histocompatibility complex
NS: noninfected spleens
ROC: receiver operating characteristic
SS: spleens of survivors
SVM: support vector machine
TOM: topological overlap matrix
TS: the tumorous spleens
