# Supplementary figures and images for: Integrated analysis of lncRNA and mRNA repertoires in Marek’s disease infected spleens identifies genes relevant to resistance
Source: BMC Genomics. 2019 Mar 28;20:245. doi: 10.1186/s12864-019-5625-1 (PMC6438004; doi:10.1186/s12864-019-5625-1)

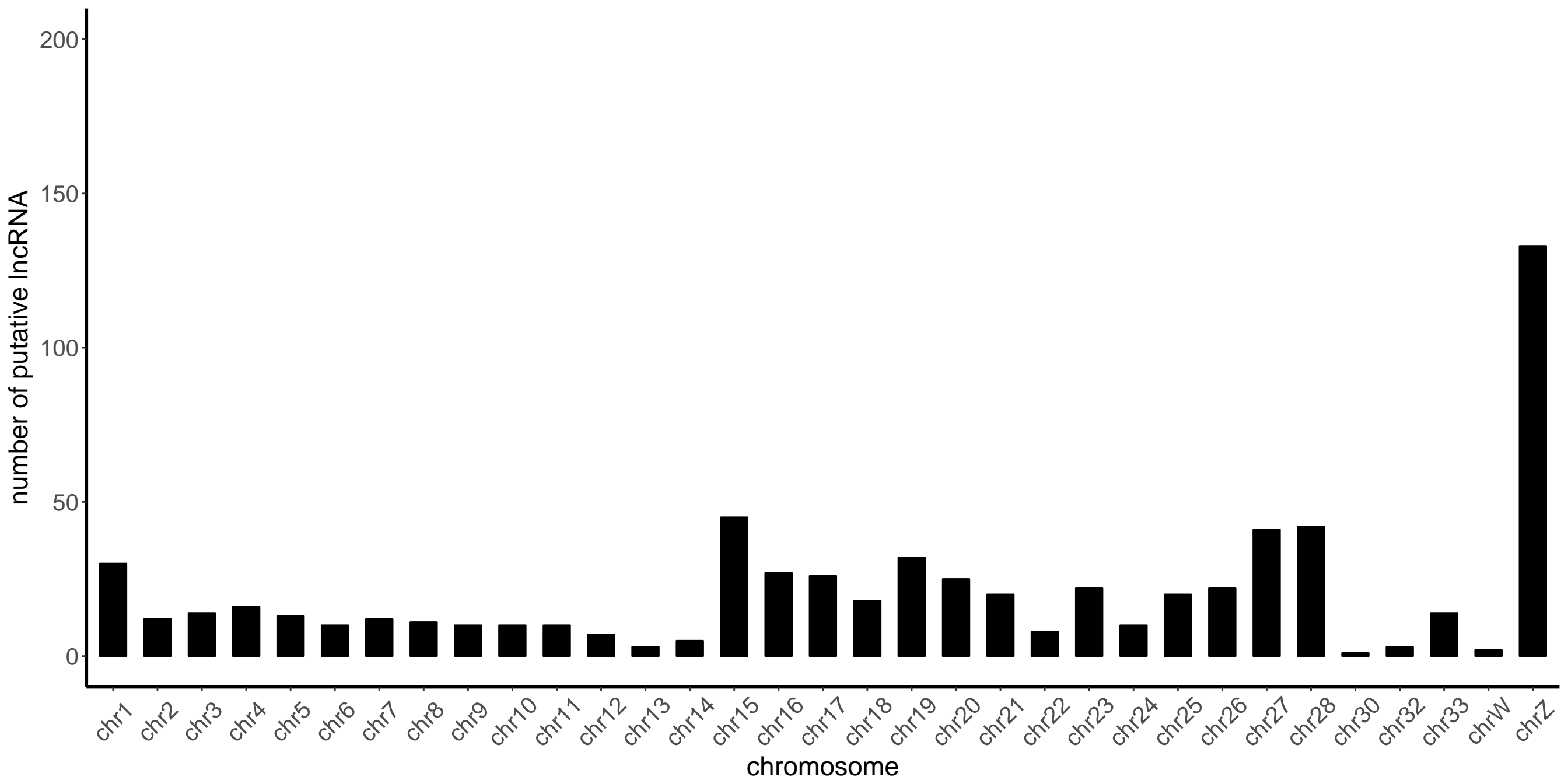

Supplement: Supplementary file 3 — Figure S1. Chromosomal distribution of 1315 putative lncRNAs. 641 putative lncRNAs (48.7%) located in small scaffolds which are not shown in this figure. Chromosome Z is the second-most putative lncRNAs which has 133 putative lncRNAs (10.1%). (PDF 5 kb) [file 12864_2019_5625_MOESM3_ESM.pdf]

A

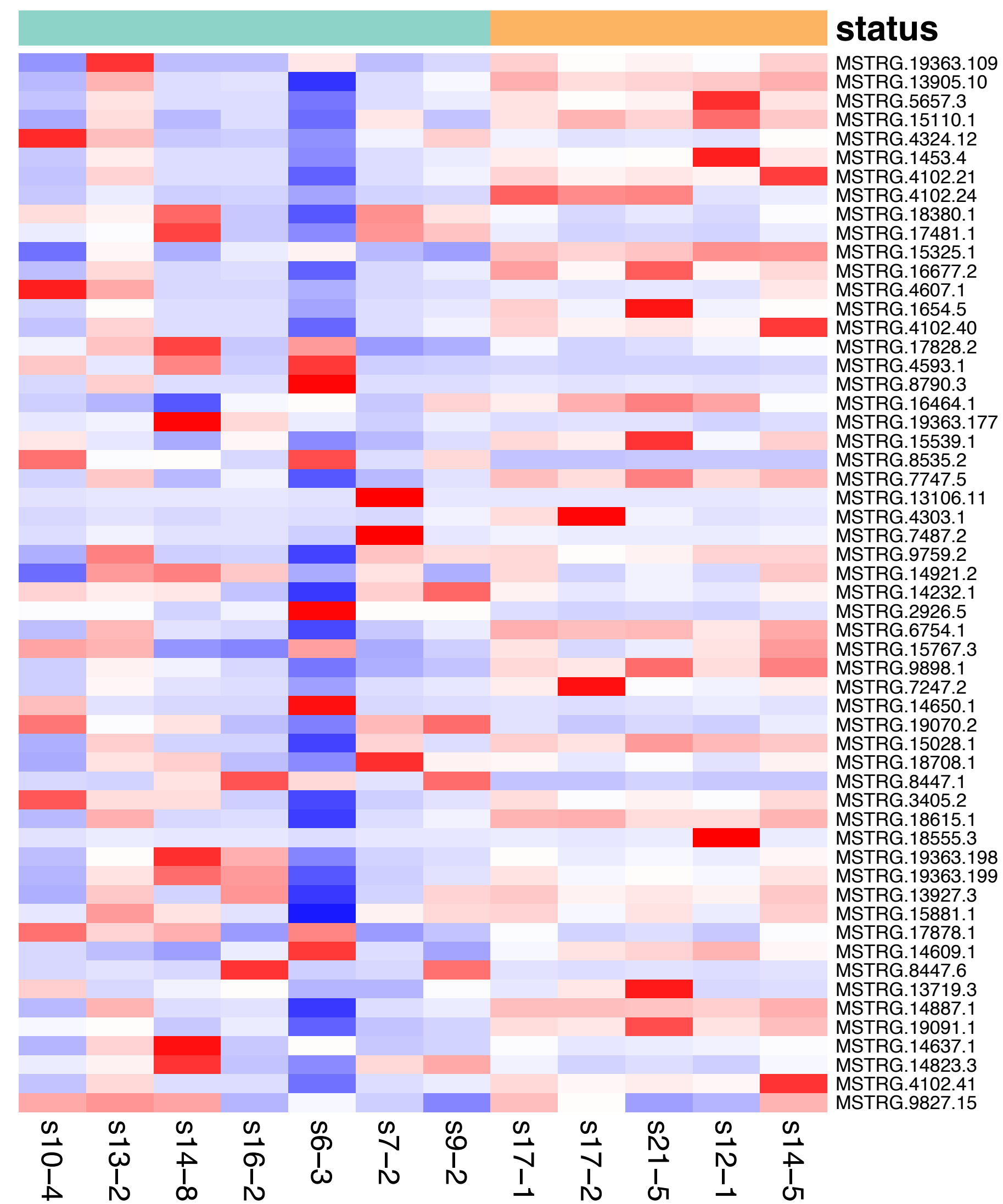

B

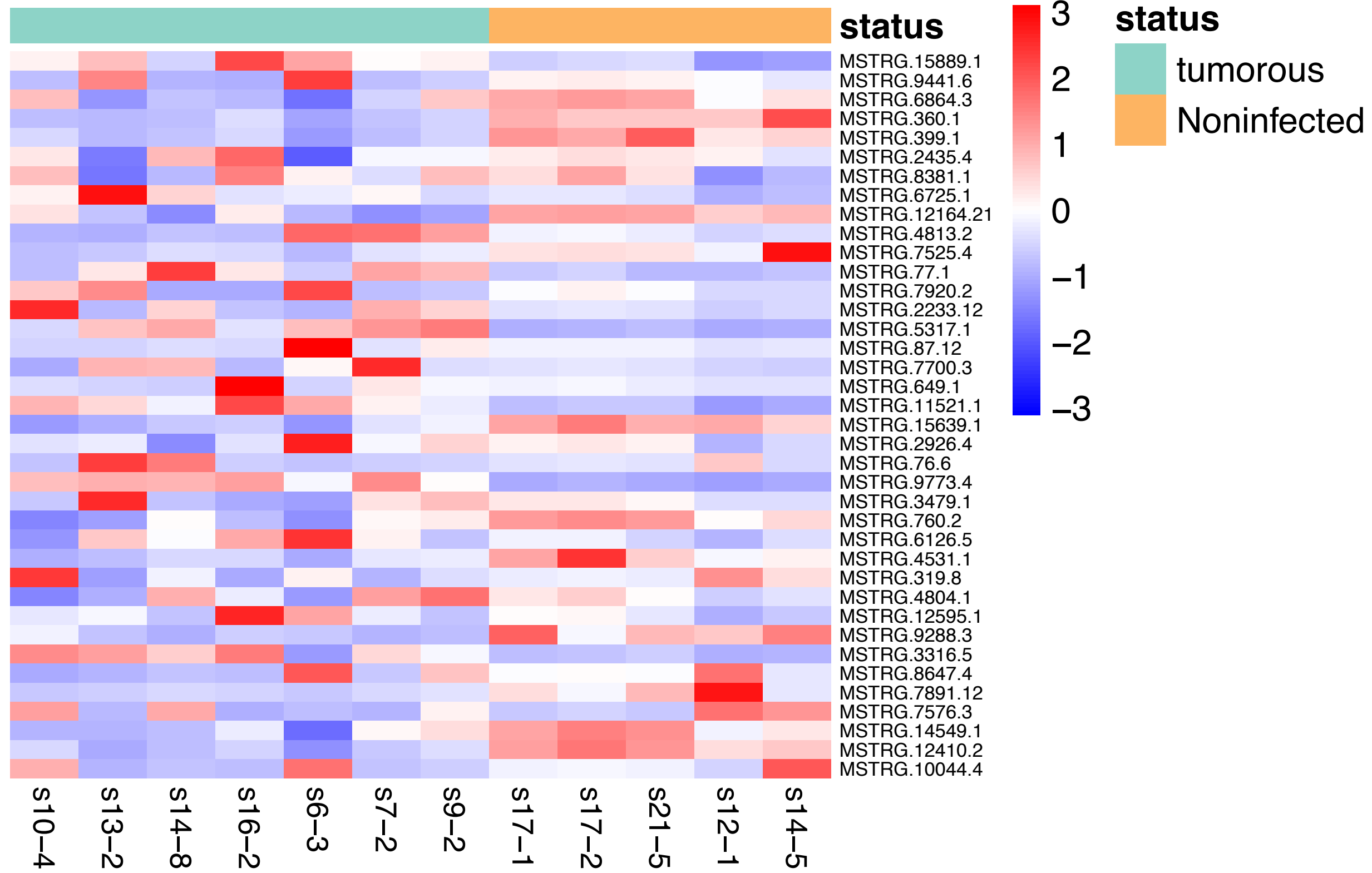

Supplement: Supplementary file 4 — Figure S2. Heatmaps of DE lncRNAs between the tumorous spleens and noninfected spleens. a the expression level of DE putative lncRNAs in each individual from the tumorous spleens and noninfected spleens. b DE known lncRNAs in each individual from the tumorous spleens and noninfected spleens. (PDF 57 kb) [file 12864_2019_5625_MOESM4_ESM.pdf]

ENSGALG00000043487

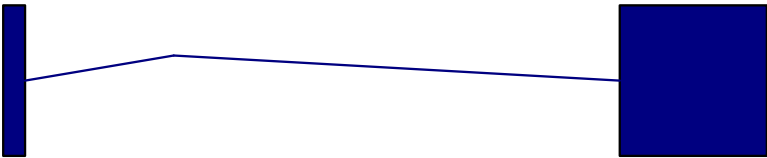

MSTRG.6754.1

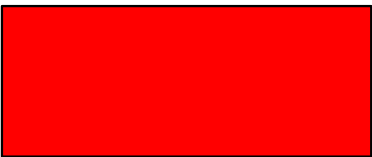

chr20

5'

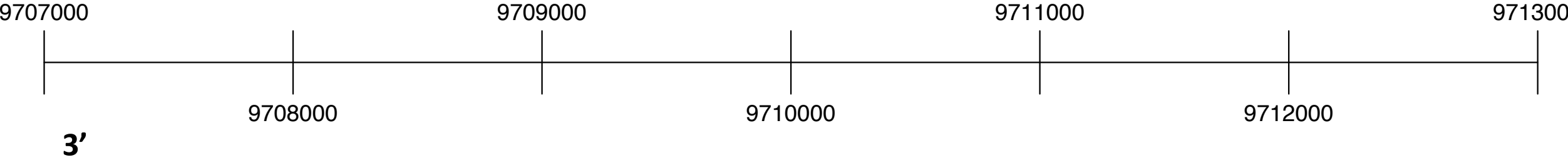

ENSGALG00000044440

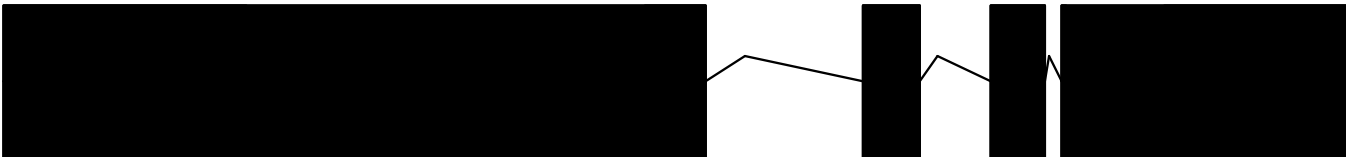

MSTRG.7747.5

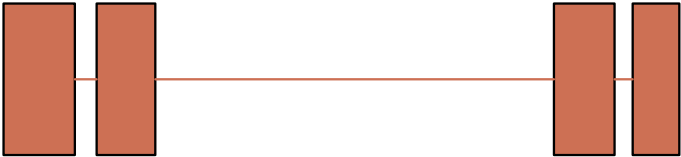

chr25

5'

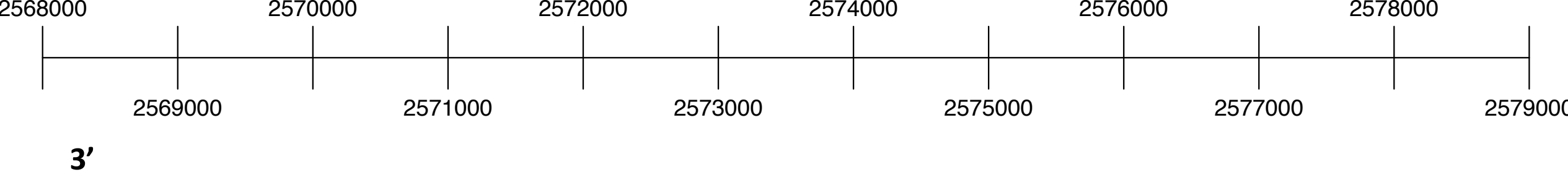

MSTRG.15539.1

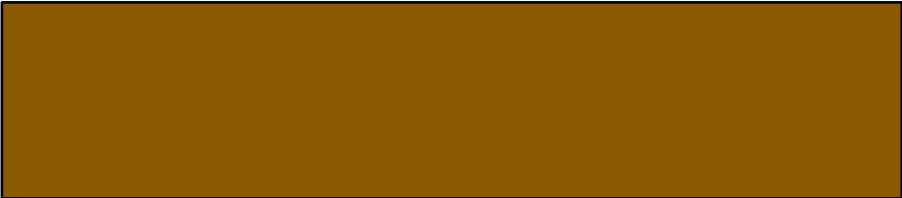

AADN04006312.1

5'

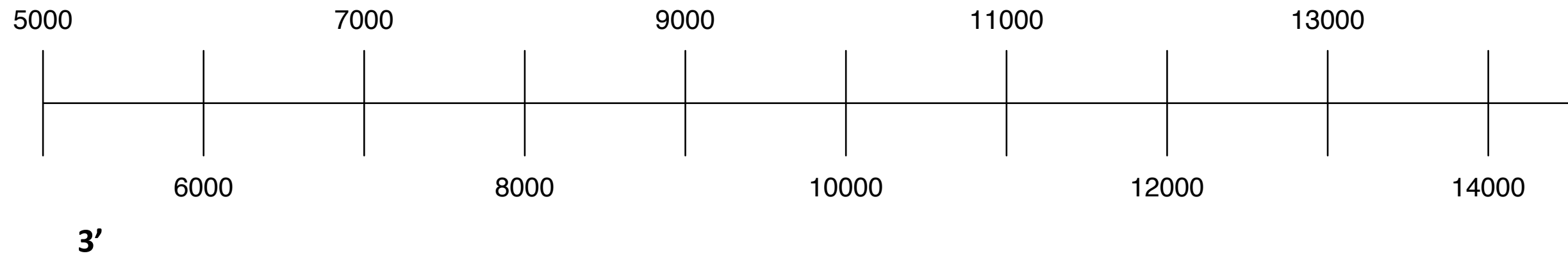

Supplement: Supplementary file 8 — Figure S3. Genomic location of three putative candidate lncRNAs and their overlapping elements. (PDF 26 kb) [file 12864_2019_5625_MOESM8_ESM.pdf]

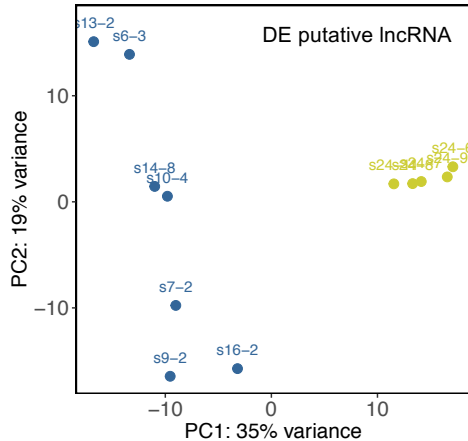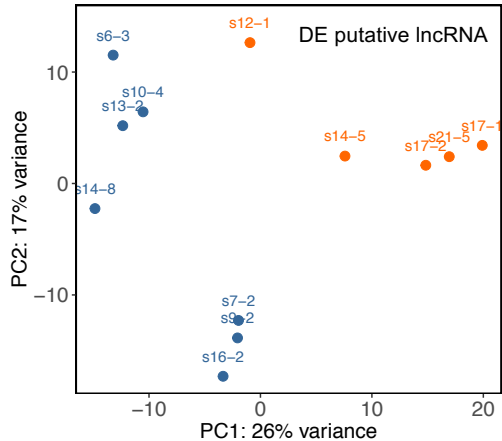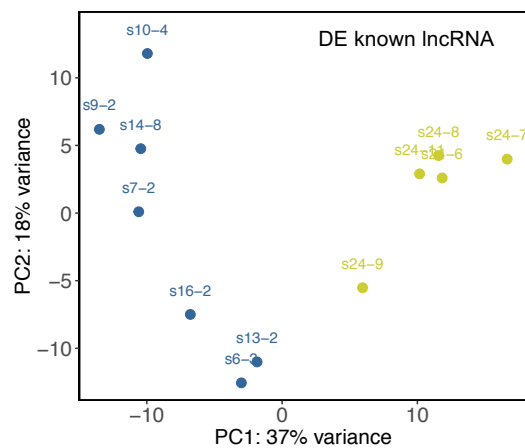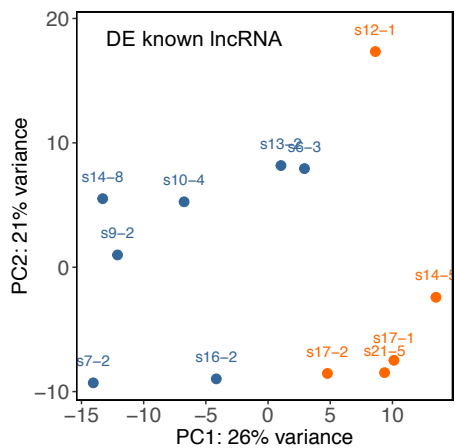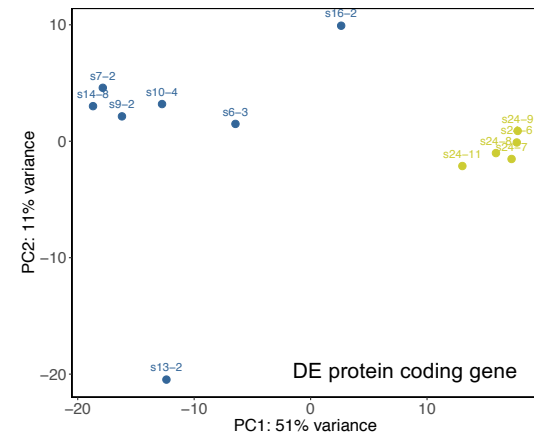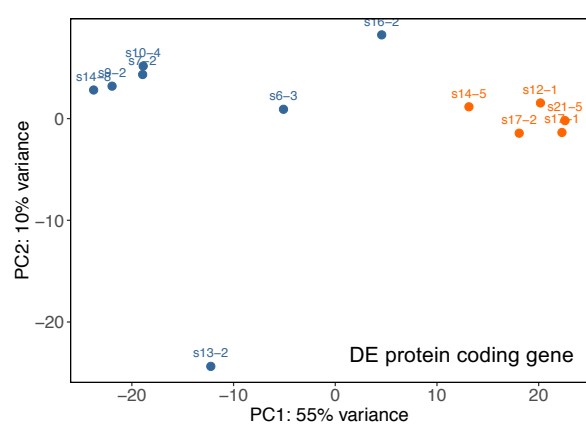

● tumorous    ● survivor    ● noninfected

Supplement: Supplementary file 10 — Figure S4. Principal components analysis (PCA) of DE protein coding genes and lncRNAs in three comparisons, including DE putative lncRNAs, DE known lncRNAs, and DE protein coding genes. (PDF 64 kb) [file 12864_2019_5625_MOESM10_ESM.pdf]
