# Supplementary material for: Integrated analysis of lncRNA and mRNA repertoires in Marek’s disease infected spleens identifies genes relevant to resistance
Source: BMC Genomics. 2019 Mar 28;20:245. doi: 10.1186/s12864-019-5625-1 (PMC6438004; doi:10.1186/s12864-019-5625-1)

Table S1. primers sequences used in quantitative PCR

| genes | direction | sequences（5’-3’） |
| --- | --- | --- |
| IGF-I | Forward | TCTTGAAGGTGAAGATGCACAC |
|  | Reverse | GCCTCCTCAGGTCACAACTC |
| MSTRG.6754.1 | Forward | AGCACCCCATAGCCTTAGGA |
|  | Reverse | CCTTTGCACAACCAGAGTGC |
| MSTRG.7747.5 | Forward | GGTGGAACAGAGCAGGGTC |
|  | Reverse | ATCAGCGATGTGCAGTTCTTC |
| MSTRG.15539.1 | Forward | GTCATTAGCCGGGCTCCAAT |
|  | Reverse | TTCGTTGGGCGCTCATTAGT |
| NONGGAT000276. | Forward | AGTCACCTTCTGACATGCGG |
|  | Reverse | ACTTGAGTCAATCTAACATGCAC |
| NONGGAT004747.2 | Forward | GAAGAGCCGCACGCTATTTG |
|  | Reverse | CAGGTGGGTTCTCCACTGTC |
| β-Actin | Forward | GAGAAATTGTGCGTGACATCA |
|  | Reverse | CCTGAAACCTCTCATTGCCA |

Gel picture of five candidate lncRNAs


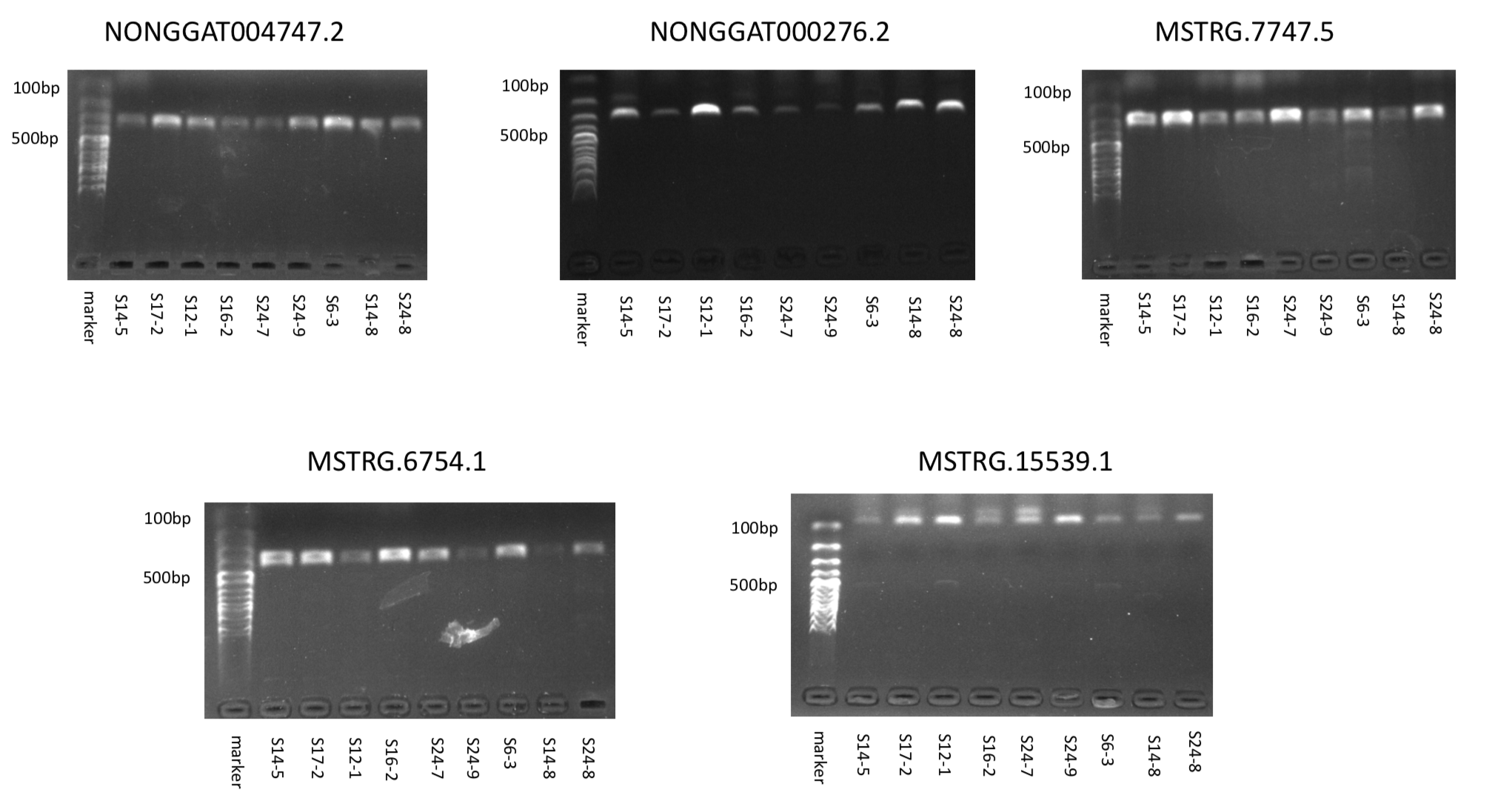

Supplement: Supplementary file 9 — Table S1. Primer sets of five candidate lncRNAs and IGF-I used in quantitative PCR analysis; QPCR gel picture of five candidate lncRNAs; (DOCX 729 kb) [file 12864_2019_5625_MOESM9_ESM.docx]
